# Sessile Droplet Profiles and Apparent Contact Angles Considering Gravity and Contact-Line Energy

**DOI:** 10.3390/mi17101204

**Published:** 2026-10-18

**Authors:** Jian Dong, Jie Wu, Wenbin Fu, Xuxuan Ai, Chengchao Pan

**Affiliations:** 1 Key Laboratory of E&M, Zhejiang University of Technology, Hangzhou 310023, China; 15240160045@163.com (J.W.); fwb20111481@163.com (W.F.); aice1225@163.com (X.A.); 2 State Key Lab of Transducer Technology, Shanghai Institute of Microsystem and Information Technology, Chinese Academy of Sciences, Shanghai 200031, China; 3 Ningbo Haitian Group Co., Ltd., Ningbo 315000, China; pcc18867@mail.haitian.com

**Keywords:** axisymmetric sessile droplet, variational model, profiles, apparent contact angles, Bashforth–Adams equation, effective contact-line energy

## Abstract

The equilibrium shape of a finite-volume axisymmetric sessile droplet is governed by surface tension, gravity and the energy associated with the three-phase contact line. A variational model is developed to predict the profiles and apparent contact angles of droplets on horizontal, macroscopically flat hydrophilic and hydrophobic surfaces. In the variational model, the free-surface variation yields the Bashforth–Adams equation for the profile, and the contact-line endpoint variation gives the transversality condition for the apparent contact angle. A numerical algorithm is developed to determine the equilibrium profiles and apparent contact angles. Experiments are conducted to calibrate and validate the model. The effective contact-line energy parameters, q, of water on the hydrophilic, ${\mathrm{SiO}}_{2}$, surface and the hydrophobic polytetrafluoroethylene, PTFE, surface are calibrated using measurements from droplets with volumes ranging from 10 to 100 μL. The calibrated, q, values are then used to predict the profiles and apparent contact angles of water droplets on the hydrophilic, ${\mathrm{SiO}}_{2}$, and hydrophobic, PTFE, surfaces with volumes of 25, 35, 55, and 75 μL, which are excluded from the calibration. Across all validation cases, the mean relative errors in the predicted apparent contact angles are 0.36% for ${\mathrm{SiO}}_{2}$ and 0.49% for PTFE, with an overall mean relative error of 0.43%. The predicted droplet profiles agree well with the experimentally extracted profiles.

## 1. Introduction

The wetting of liquid droplets on solid surfaces is important in coating, printing, microfluidics, semiconductor cleaning, biosensing, and surface modification. The apparent contact angle is commonly used to characterize the macroscopic wetting state. The complete free-surface profile provides further information about droplet spreading and equilibrium morphology. For the microliter droplets considered in this study, gravitational deformation cannot always be neglected. Their equilibrium shapes depend on the interfacial properties of the liquid and solid materials, droplet volume, gravity, and the state of the three-phase contact line [1,2].

Young’s equation describes the local balance of the interfacial tensions at the three-phase contact line on an ideally smooth, chemically homogeneous, and rigid solid surface [3]. The Young–Laplace equation relates the pressure difference across a curved interface to its mean curvature [4]. For an axisymmetric sessile droplet under gravity, the governing equation for the profile can be written in the Bashforth–Adams form [5,6,7,8,9,10,11]. However, the position of the contact line still requires an appropriate boundary condition. For example, axisymmetric drop shape analysis (ADSA) is an established inverse method that infers interfacial properties by fitting theoretical profile solutions to measured droplet shapes or dimensions [6,7]. Predicting the apparent contact angle and complete free-surface profile at volumes excluded from calibration, however, requires a contact-line boundary condition that remains applicable across the investigated size range.

Experimental substrates are not ideally homogeneous surfaces. Cutting, polishing, oxidation, cleaning, coating, and film attachment can leave microscopic roughness, chemical heterogeneity, adsorbed contamination, and local defects. These features may cause contact-line pinning and contact-angle hysteresis [12,13]. Measured contact angles are also affected by droplet deposition, sample preparation, equilibration time, baseline identification, and image resolution [14,15,16,17,18]. Water contact angles reported for silicon and silicon oxide vary with the oxidation state, cleaning procedure, and surface contamination [19]. The angle obtained from a side-view image is, therefore, treated in this study as an apparent contact angle associated with the current surface state and experimental conditions.

Microscopic roughness and chemical heterogeneity create an uneven free-energy landscape near the three-phase contact line. Eral et al. [12] defined contact-angle hysteresis as the difference between the advancing and receding contact angles and discussed the metastable states within this interval. Energy barriers separate these states and may prevent the droplet from reaching the global free-energy minimum. Butt et al. [13] related contact-angle hysteresis to nanoscopic surface structures that pin the contact line. Joanny and de Gennes [20] showed that weak heterogeneity may distort the contact line without causing hysteresis. A strong localized defect, however, can produce two stable contact-line positions. Hysteresis appears when the defect strength exceeds a threshold value. Molecular-kinetic theory explains contact-line motion from a dynamic perspective. In this theory, molecules move between neighboring sites through thermally activated processes. Sedev [21] related the required free-energy barrier to adhesion at the solid surface. The additional contact-line contribution is represented phenomenologically by qL, where L is the contact-line length and q is an effective contact-line energy parameter. Its value is calibrated from static apparent contact angles and contact-line radii for the prepared substrate under the specified deposition protocol.

Boruvka and Neumann [22] extended classical capillarity by treating the three-phase contact line as a thermodynamic entity with a line free energy. When the line energy per unit length is constant, the total contact-line energy is proportional to the contact-line length. For an axisymmetric droplet with contact-line radius $x_{k}$, $L=2\pi x_{k}$, and $E_{l}=\;qL=2\pi qx_{k}$. Bormashenko [23] later studied wetting within a variational framework. The Young and Boruvka–Neumann equations were shown to follow from the transversality condition at a movable contact line. These results support the use of qL in the total free energy and place its effect in the contact-line boundary condition. In the present model, variation in the free surface yields the gravity-dependent Bashforth–Adams equation. Variation in the contact-line endpoint gives the contact-line boundary condition:

(1) $$\cos\theta_{k}=\cos\theta_{\infty }-\frac{q}{\gamma_{lv}x_{k}}$$

where $\theta_{k}$ is the apparent contact angle, $\theta_{\infty }$ is the reference contact angle and $\gamma_{lv}$ is the liquid–gas interfacial tension. For a constant line-energy coefficient, Equation (1) has the classical Boruvka–Neumann form [22,23]. The parameter q does not appear directly in the curvature equation of the free surface. It changes the contact-line radius and apparent contact angle through the transversality condition. The resulting shift in the contact-line position also changes the complete equilibrium droplet profile.

This study evaluates a prescribed-volume prediction procedure that combines the gravity-dependent Bashforth–Adams equation, an effective contact-line boundary condition, and a volume constraint. For water on prepared ${\mathrm{SiO}}_{2}$ and PTFE substrates, one pair of $\theta_{\infty }$ and q is calibrated using measurements at nine selected volumes and then held fixed for predictions at 25, 35, 55, and 75 μL. The assessment compares apparent contact angles with a separately calibrated q = 0 baseline and examines the associated droplet profiles. The contribution is an experimental evaluation of this parameter-fixed prediction procedure for the tested substrates and deposition protocol, using established capillary relations.

## 2. Theoretical Models

### 2.1. Geometric Model and Total Free Energy

Consider a static incompressible droplet resting on a horizontal rigid substrate. Under the combined effects of gravity and surface tension, the droplet is assumed to attain an approximately axisymmetric equilibrium configuration. A Cartesian coordinate system is introduced with the droplet apex taken as the origin, O. The x-axis points horizontally to the right, and the z-axis points vertically downward. The right-side meridional profile of the droplet is described by

(2) $$x=x(z),\;\;0\leq z\leq z_{k}$$

Here, $z_{k}$ is the vertical distance from the apex to the substrate, and $o_{k}$ is the center of the three-phase contact circle. The radius of the three-phase contact circle is defined as

(3) $$x_{k}=x\left(z_{k}\right)$$

**Figure 1:**
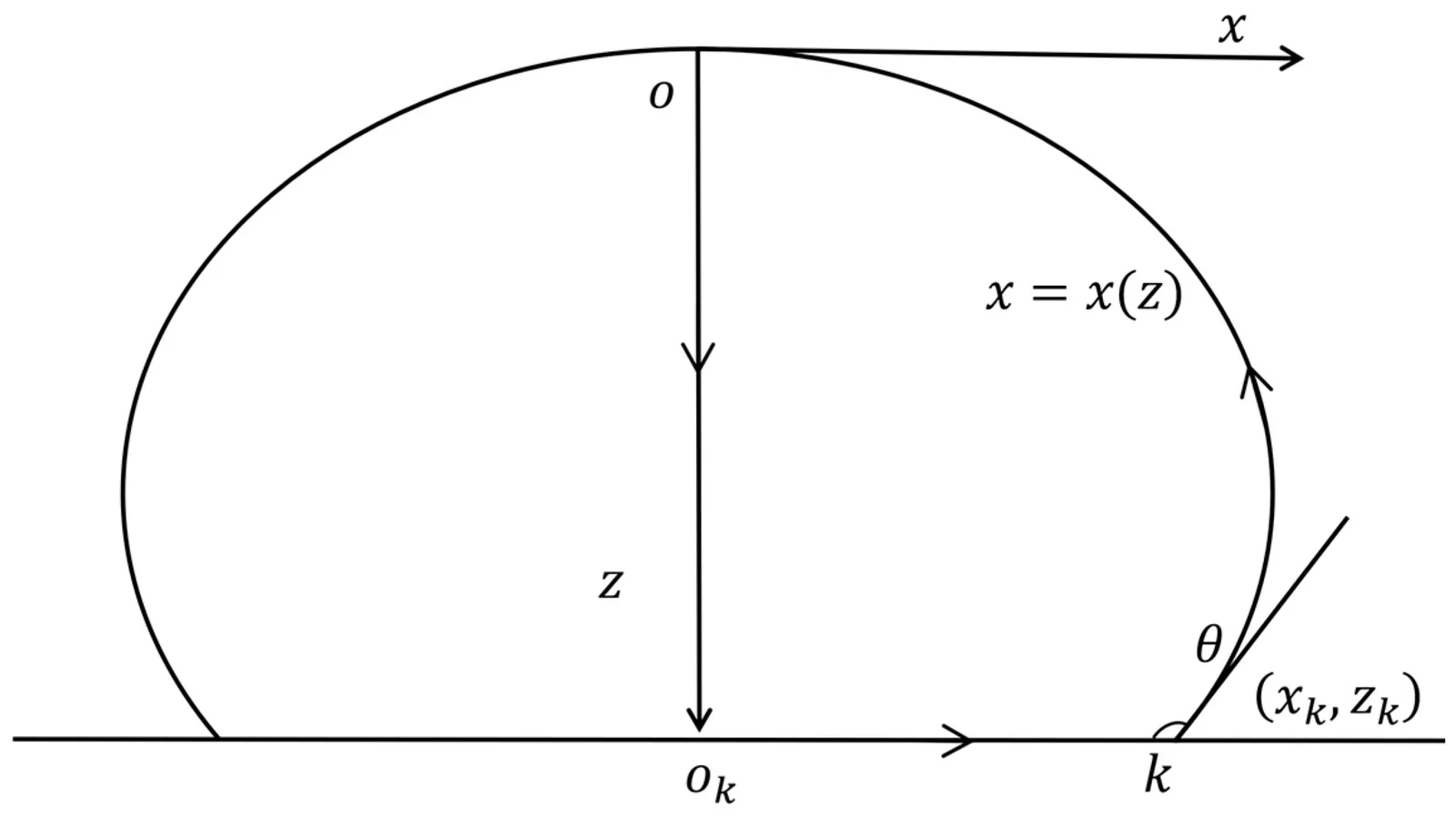
Geometric model of an axisymmetric droplet on a horizontal substrate.

The geometric configuration is illustrated in Figure 1.

The following assumptions are adopted. The droplet is incompressible and remains in static equilibrium. Evaporation, airflow disturbances, temperature gradients, and inertial effects are neglected. For a given solid–liquid–gas system, the reference contact angle, $\theta_{\infty }$, is given by Young’s equation as follows:

(4) $$\cos\theta_{\infty }=\frac{\gamma_{sv}-\gamma_{sl}}{\gamma_{lv}}$$

Here, $\gamma_{sl}$ and $\gamma_{sv}$ are the solid–liquid and solid–vapor interfacial tensions, respectively. The effective contact-line energy parameter, q, is the coefficient of the linear energy contribution, $E_{l}=qL$, and has units of J/m. It is assumed constant for each prepared substrate over the investigated droplet-size range. Its calibrated value characterizes the apparent static contact-line response under the specified deposition protocol. Contact-angle-based estimates can involve multiple line and interfacial contributions [24]. The present measurements do not establish q as an independently measured microscopic line tension or activation barrier.

The total free energy of the droplet, E, consists of the gravitational potential energy, $E_{g}$, the liquid–vapor interfacial energy, $E_{e-s}$, the net solid interfacial energy, $E_{i-s}$, and the effective contact-line excess free energy, $E_{l}$.

(5) $$E=E_{g}+E_{e-s}+E_{i-s}+E_{l}$$

For a prescribed droplet volume, V, each energy contribution is calculated from the droplet profile.

The substrate plane is taken as the reference level for the gravitational potential energy. Let $\left(x_{in},z_{in}\right)$ be an arbitrary point in the meridional cross-section of the droplet. The gravitational potential energy, $E_{g}$, is expressed as

(6) $$\begin{array}{l} E_{g}=\left(\rho_{l}-\rho_{v}\right)g\iint_{D}\left(z_{k}-z_{in}\right)2\pi x_{in}dx_{in}dz_{in}\\ =2\pi \left(\rho_{l}-\rho_{v}\right)g\left(\iint_{D}z_{k}x_{in}dx_{in}dz_{in}-\iint_{D}x_{in}z_{in}dx_{in}dz_{in}\right) \end{array}$$

where D is the meridional cross-sectional domain of the droplet, $\rho_{l}$ is the liquid density and $\rho_{v}$ is the vapor density. We use Green’s formula to convert the integral within the meridional plane of the droplet into an integral along the droplet profile. Setting $Q_{1}=\frac{1}{2}{x_{in}}^{2}$, we obtain

(7) $$\iint_{D}\frac{\partial Q_{1}}{\partial x_{in}}dx_{in}dz_{in}=\iint_{D}x_{in}dx_{in}dz_{in}=\oint_{\Gamma }Q_{1}\left(x,z\right)dz=\oint_{\Gamma }\frac{1}{2}x^{2}dz=\int_{\hat{ko}}\frac{1}{2}x^{2}dz=\int_{0}^{z_{k}}\frac{1}{2}x^{2}dz$$

where $\Gamma =\hat{ko}+\vec{oo_{k}}+\vec{o_{k}k}$. Then, $\iint_{D}z_{k}x_{in}dx_{in}dz_{in}=\int_{0}^{z_{k}}\frac{1}{2}z_{k}x^{2}dz$. Setting $Q_{2}=\frac{1}{2}{x_{in}}^{2}z_{in}$ gives

(8) $$\iint_{D}\frac{\partial Q_{2}}{\partial x_{in}}dx_{in}dz_{in}=\iint_{D}x_{in}z_{in}dx_{in}dz_{in}=\oint_{\Gamma }\frac{1}{2}x^{2}zdz=\int_{\hat{ko}}\frac{1}{2}x^{2}zdz=\int_{0}^{z_{k}}\frac{1}{2}x^{2}zdz$$

Substituting Equations (7) and (8) into Equation (6) gives

(9) $$E_{g}=2\pi \left(\rho_{l}-\rho_{v}\right)g\left(\int_{0}^{z_{k}}\frac{1}{2}z_{k}x^{2}dz-\int_{0}^{z_{k}}\frac{1}{2}x^{2}zdz\right)=\pi \left(\rho_{l}-\rho_{v}\right)g\int_{0}^{z_{k}}\left(z_{k}x^{2}-x^{2}z\right)dz$$

The remaining energy contributions are given by

(10) $$E_{e-s}=\int_{0}^{z_{k}}2\pi \gamma_{lv}x{\left(1+{x^{\prime }}^{2}\right)}^{\frac{1}{2}}dz,x^{\prime }=\frac{dx}{dz}$$

(11) $$E_{i-s}=\pi {x_{k}}^{2}\left(\gamma_{sl}-\gamma_{sv}\right)$$

and

(12) $$E_{l}=q\cdot 2\pi x_{k}$$

Substituting Equations (9)–(12) into Equation (5) yields the total energy functional:

(13) $$E\left(z,x,x^{\prime }\right)=\int_{0}^{z_{k}}\left[\pi \left(\rho_{l}-\rho_{v}\right)g\left(z_{k}x^{2}-x^{2}z\right)+2\pi \gamma_{lv}x{\left(1+{x^{\prime }}^{2}\right)}^{\frac{1}{2}}\right]dz+\pi {x_{k}}^{2}\left(\gamma_{sl}-\gamma_{sv}\right)+q\cdot 2\pi x_{k}$$

The volume constraint is as follows:

(14) $$V=\int_{0}^{z_{k}}\pi x^{2}\left(z\right)dz$$

The auxiliary functional $\Pi \left(z,x,x^{\prime }\right)$ is then written as

(15) $$\begin{array}{l} \Pi \left(z,x,x^{\prime }\right)=\int_{0}^{z_{k}}\left[\pi \left(\rho_{l}-\rho_{v}\right)g\left(z_{k}x^{2}-x^{2}z\right)+2\pi \gamma_{lv}x{\left(1+{x^{\prime }}^{2}\right)}^{\frac{1}{2}}+\lambda \pi x^{2}\left(z\right)\right]dz+\\ \pi {x_{k}}^{2}\left(\gamma_{sl}-\gamma_{sv}\right)+q\cdot 2\pi x_{k}=\int_{0}^{z_{k}}F\left(z,x,x^{\prime }\right)dz+\Phi \left(x_{k}\right) \end{array}$$

where

(16) $$F\left(z,x,x^{\prime }\right)=\pi \left(\rho_{l}-\rho_{v}\right)g\left(z_{k}x^{2}-x^{2}z\right)+2\pi \gamma_{lv}x{\left(1+{x^{\prime }}^{2}\right)}^{\frac{1}{2}}+\lambda \pi x^{2}$$

And

(17) $$\Phi \left(x_{k}\right)=\pi {x_{k}}^{2}\left(\gamma_{sl}-\gamma_{sv}\right)+q\cdot 2\pi x_{k}$$

### 2.2. Droplet Profiles Considering Gravity and Contact-Line Energy

Applying the Euler–Lagrange equation to Equation (16) gives the governing equation for the droplet profile.

Because $F_{x}-\frac{d}{dz}F_{x^{\prime }}=0$, $F_{x}=2\pi x\left(\rho_{l}-\rho_{v}\right)g\left(z_{k}-z\right)+2\pi \gamma_{lv}{\left(1+{x^{\prime }}^{2}\right)}^{\frac{1}{2}}+2\lambda \pi x$ and $F_{x^{\prime }}=2\pi \gamma_{lv}xx^{\prime }{\left(1+{x^{\prime }}^{2}\right)}^{-\frac{1}{2}}$, we obtain

(18) $$-\frac{x^{\prime \prime }}{{\left(1+{x^{\prime }}^{2}\right)}^{3/2}}+\frac{1}{x{\left(1+{x^{\prime }}^{2}\right)}^{1/2}}=\frac{\left(\rho_{l}-\rho_{v}\right)g}{\gamma_{lv}}\left(z-z_{k}\right)-\frac{\lambda }{\gamma_{lv}}$$

Since the droplet surface is smooth and axisymmetric, the apex is an umbilic point, where the two principal radii of the curvature are equal. Denoting their common value by b>0, we obtain $\frac{2}{b}=-\left(\frac{\left(\rho_{l}-\rho_{v}\right)g}{\gamma_{lv}}z_{k}+\frac{\lambda }{\gamma_{lv}}\right)$. Expressing the right-hand droplet profile as $z=z\left(x\right)$ in the zox coordinate system, we obtain the following differential equation:

(19) $$\frac{z^{\prime \prime }}{{\left(1+{z^{\prime }}^{2}\right)}^{\frac{3}{2}}}+\frac{z^{\prime }}{x{\left(1+{z^{\prime }}^{2}\right)}^{\frac{1}{2}}}=\frac{2}{b}+\frac{\left(\rho_{l}-\rho_{v}\right)g}{\gamma_{lv}}z$$

Here, z′=dz/dx and $z\prime \prime =d^{2}z/dx^{2}$. The fixed conditions are z(0)=0 and z′(0)=0. Equation (19) retains gravity in dimensional form. Scaling the coordinates by the apex radius of curvature b yields the Bond number $Bo_{b}=(\rho_{l}-\rho_{v})gb^{2}/\gamma_{lv}$ [25].

Thus, Equation (19) is the Bashforth–Adams equation, and the tangent angle of the profile is given by Equation (20).

(20) $$\theta =\arccos\frac{x^{\prime }}{\sqrt{1+{x^{\prime }}^{2}}}$$

### 2.3. Apparent Contact Angles Considering Gravity and Contact-Line Energy

Using the endpoint energy term $\Phi \left(x_{k}\right)$ given in Equation (17), we obtain the boundary conditions from the transversality conditions of the Bolza problem as follows:

(21) $${\left.F_{x^{\prime }}\right|}_{z=z_{0}}=\frac{\partial \Phi \left(x_{k}\right)}{\partial x_{0}}$$

(22) $${\left.F_{x^{\prime }}\right|}_{z=z_{k}}=-\frac{\partial \Phi \left(x_{k}\right)}{\partial x_{k}}$$

At the apex, $x_{0}\equiv 0$, giving ${\left.F_{x^{\prime }}\right|}_{z=z_{0}}=2\pi \gamma_{lv}x_{0}x_{0}^{\prime }{\left(1+{\left(x_{0}^{\prime }\right)}^{2}\right)}^{-\frac{1}{2}}\equiv 0$. Since the endpoint energy $\Phi \left(x_{k}\right)$ is independent of $x_{0}$, $\frac{\partial \Phi \left(x_{k}\right)}{\partial x_{0}}\equiv 0$. Both sides of Equation (21), therefore, vanish identically, and the condition is automatically satisfied. Only Equation (22) needs to be considered. Substituting ${\left.F_{x^{\prime }}\right|}_{z=z_{k}}=2\pi \gamma_{lv}x_{k}x_{k}^{\prime }{\left(1+{x_{k}^{\prime }}^{2}\right)}^{-\frac{1}{2}}$ and $\frac{\partial \Phi \left(x_{k}\right)}{\partial x_{k}}=2\pi x_{k}\left(\gamma_{sl}-\gamma_{sv}\right)+2\pi q$ into Equation (22) gives the contact-line boundary condition:

(23) $$\gamma_{lv}x_{k}x_{k}^{\prime }{\left(1+{x_{k}^{\prime }}^{2}\right)}^{-\frac{1}{2}}=-\left[x_{k}\left(\gamma_{sl}-\gamma_{sv}\right)+q\right]$$

Combining Equation (20) with the contact-line condition in Equation (23) gives the apparent contact angle as

(24) $$\theta_{k}=\arccos\frac{x_{k}^{\prime }}{\sqrt{1+{x_{k}^{\prime }}^{2}}}$$

The transversality condition describes a stationary contact line. Finite-speed contact-line motion requires a dynamic boundary relation accounting for dissipative effects [21]. The effective energy parameter, q, does not specify a contact-line friction coefficient.

## 3. Numerical Methods

### 3.1. Calibration of the Effective Contact-Line Energy Parameter

Combining Equations (4), (23), and (24) gives the contact-line condition in Equation (1). The effective contact-line energy parameter, q, and $\theta_{\infty }$ are calibrated jointly from the measured apparent contact angles, $\theta_{k}$, and contact-line radii, $x_{k}$, using Equation (1).

(25) $$\left({\hat{\theta }}_{\infty },\hat{q}\right)=\underset{\theta_{\infty },q}{\arg\min}\sum_{i=1}^{n}{\left[\cos\theta_{ki}-\cos\theta_{\infty }+\frac{q}{\gamma_{lv}x_{ki}}\right]}^{2}$$

where $\theta_{ki}$ and $x_{ki}$ denote the measured apparent contact angle and contact-line radius of the ith calibration droplet, respectively, with i=1,…,n. The parameters $\theta_{\infty }$ and q are determined using the nlinfit function in MATLAB R2023b.

### 3.2. Determination of Profiles and Apparent Contact Angles

After q and $\theta_{\infty }$ are determined, the droplet profile and apparent contact angle are calculated using the following procedure. The calculation starts from a prescribed value of b, from which the corresponding B-A profile is obtained. Subsequently, we search along the B-A profile from the apex (z=0) until reaching the position $z=z_{k}$ at which $x_{k}$ and $x_{k}^{\prime }$ satisfy the contact-line condition given in Equation (23). Once $z_{k}$ is determined, Equation (14) is integrated with respect to z from 0 to $z_{k}$ to calculate the droplet volume $V_{\left(b,z_{k}\right)}$. $V_{\left(b,z_{k}\right)}$ is compared with the given droplet volume, V. If the relative volume error exceeds $10^{-4}$, b is increased by Δb, and the calculation is repeated until the prescribed tolerance is satisfied. The resulting B-A profile and $\theta_{k}$ are recorded as the predicted droplet profile and apparent contact angle.

The numerical procedure is summarized in Figure S1 in the Supplementary Materials.

## 4. Experiments

Deionized water is used as the test liquid. At room temperature, its density and liquid–gas interfacial tension are taken as $\rho_{l}=1000\;{\mathrm{kg}/m}^{3}$ and $\gamma_{lv}=0.072\;N/m$. Macroscopically flat ${\mathrm{SiO}}_{2}$ and PTFE surfaces are selected to represent different wetting conditions. Before each experiment, the ${\mathrm{SiO}}_{2}$ samples are cleaned with absolute ethanol and deionized water and then dried with nitrogen. The PTFE films are cleaned with absolute ethanol and allowed to dry. Each film is placed flat on the horizontal sample stage to reduce errors caused by warping or local wrinkles. The substrates are treated as planar at the droplet scale. The microscopic surface topography was not characterized.

**Figure 2:**
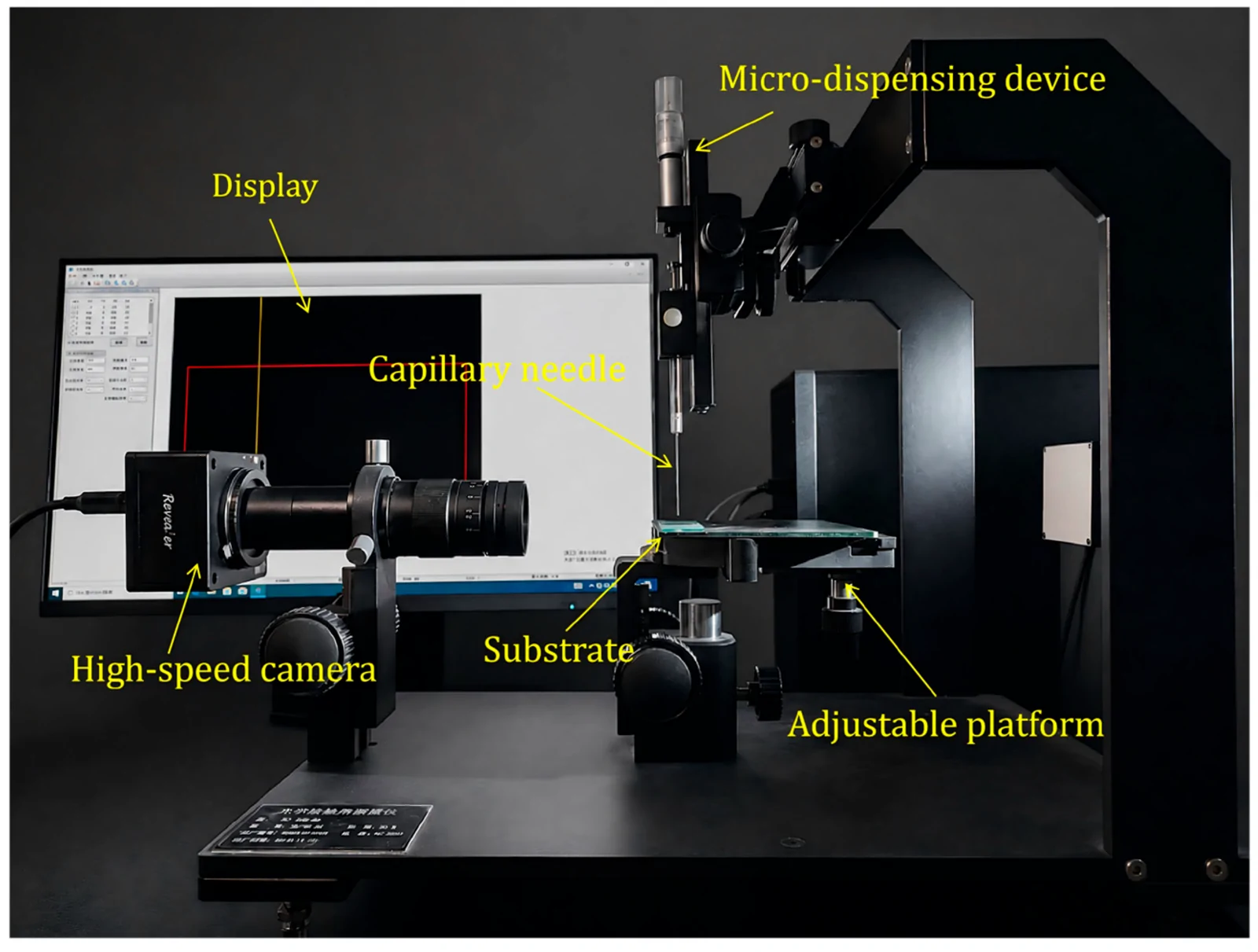
Experimental setup for static droplet measurement and side-view imaging.

Static droplet measurements are carried out using an SDC-80 contact-angle goniometer (Sindin, Dongguan, China). The experimental setup consists of a horizontal sample stage, a micro-dispensing device, a side-view imaging system and a backlight source, as shown in Figure 2. Each substrate is leveled before a prescribed volume of deionized water is deposited with a microsyringe. The needle-to-substrate distance and dispensing rate are controlled to limit droplet oscillation and contact-line motion during deposition. Side-view images are recorded after the droplet reaches a stable state. Five independent measurements are conducted for each droplet volume, and the results are reported as the mean ± standard deviation.

The tested droplet volumes are divided into a calibration set and a validation set. The calibration set includes droplet volumes of 10, 15, 20, 30, 40, 50, 70, 85, and 100 μL. The measured contact-line radii and apparent contact angles are used to calibrate q and $\theta_{\infty }$ for each substrate. Side-view images are processed by edge detection and baseline identification. Pixel coordinates are converted into physical coordinates using the calibrated image scale and expressed in the coordinate system defined in Section 2.1. The droplet profile, the left and right apparent contact angles, $\theta_{L}$ and $\theta_{R}$, and the contact-line diameter, D, are determined from each image. The apparent contact angle, $\theta_{k}$, and the contact-line radius, $x_{k}$, are calculated as

(26) $$\theta_{k}=\frac{\theta_{L}+\theta_{R}}{2},x_{k}=\frac{D}{2}$$

The validation set consists of droplets of 25, 35, 55, and 75 μL, which are not included in the calibration set. Since all validation volumes fall within the calibration range, the validation examines the predictive performance of the model within this range.

The validation concerns static apparent contact angles and droplet profiles under the stated deposition protocol. Advancing and receding contact angles were not measured, so contact-angle hysteresis and wetting-cycle dissipation are not quantified.

## 5. Results and Discussion

### 5.1. Calibration Results for $\theta_{\infty }$ and q

The contact line radius, $x_{k}$, and the apparent contact angle, $\theta_{k}$, for the calibration droplets on the ${\mathrm{SiO}}_{2}$ and PTFE surfaces are summarized in Table 1. The fitting procedure yields $\theta_{\infty ,{\mathrm{SiO}}_{2}}=56.71^{{}^{\circ}}$ and $q_{{\mathrm{SiO}}_{2}}=2.169\times 10^{-5}\;N$ for water on the ${\mathrm{SiO}}_{2}$ surface. For the PTFE surface, $\theta_{\infty ,\mathrm{PTFE}}=90.85^{{}^{\circ}}$ and $q_{\mathrm{PTFE}}=2.135\times 10^{-5}\;N$.

### 5.2. Comparison of Predicted and Experimental Apparent Contact Angles

For comparison, a baseline with q=0 is evaluated using the same calibration and validation sets. The gravity-dependent Bashforth–Adams equation and the prescribed-volume constraint are retained. Setting q=0 in Equation (1) results in a volume-independent contact angle. For each substrate, the constant reference angle is fitted by minimizing the squared residuals in the cosine space over the nine calibration-volume mean angles. The fitted angle is the inverse cosine of their mean cosine: 63.09° for ${\mathrm{SiO}}_{2}$ and 98.05° for PTFE. The validation measurements are excluded from this estimate.

**Figure 3:**
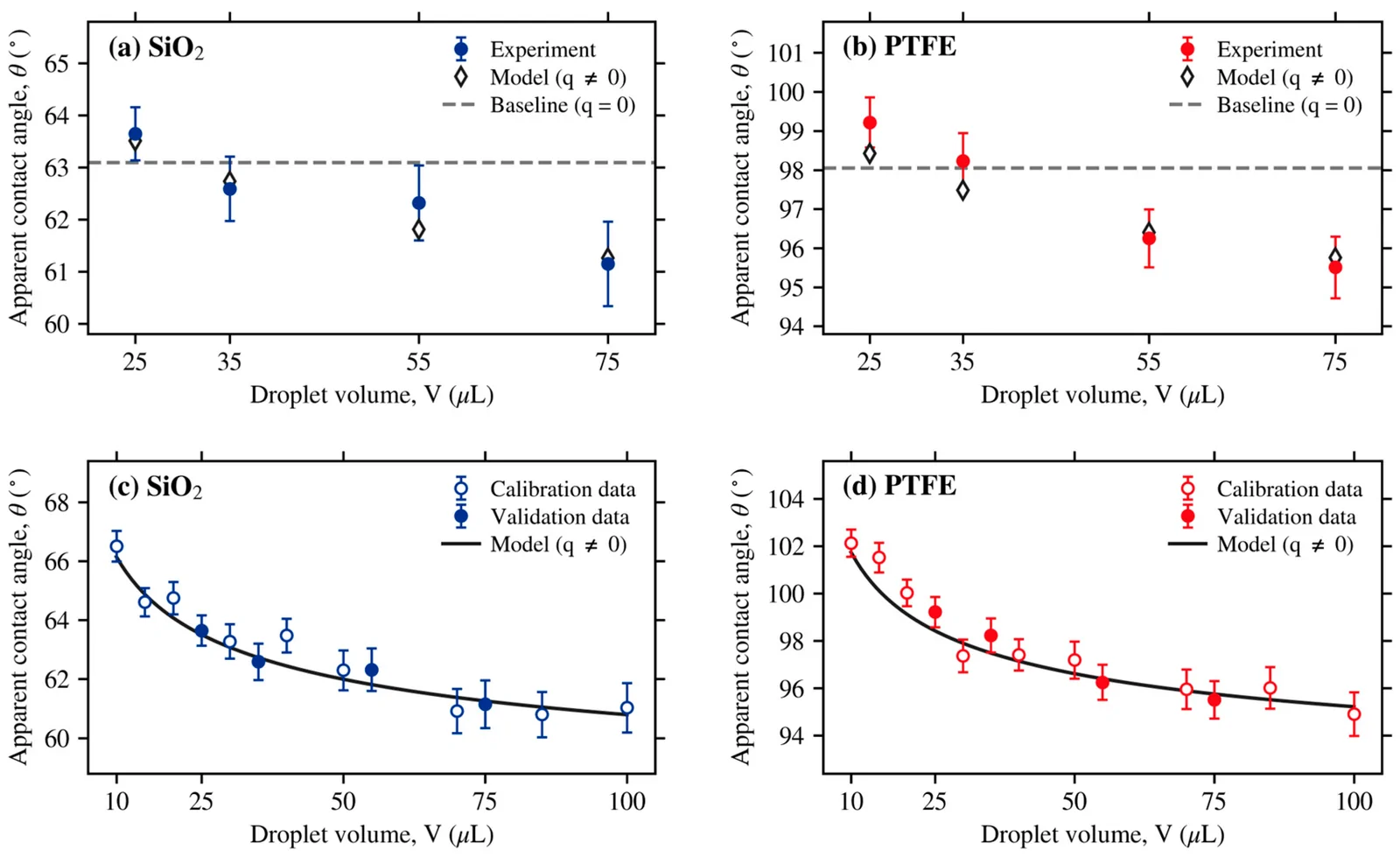
Experimental apparent contact angles and model predictions for water droplets on the ${\mathrm{SiO}}_{2}$ and PTFE surfaces. (**a**,**b**) Validation results at 25, 35, 55, and 75 μL on ${\mathrm{SiO}}_{2}$ and PTFE, respectively. Filled circles represent experimental measurements, and open diamonds represent predictions of the model with q≠0. Gray dashed lines indicate the constant-angle q=0 baseline. (**c**,**d**) Apparent contact angle variation over 10–100 μL on ${\mathrm{SiO}}_{2}$ and PTFE, respectively. Open and filled circles denote calibration and validation measurements, respectively. Solid curves represent model predictions calculated with q and $\theta_{\infty }$ held fixed after calibration. Experimental points are the means of five independent measurements at each volume, and error bars indicate ± one standard deviation.

Figure 3a,b compare the experimental apparent contact angles with the predictions of the present model and the q=0 baseline at 25, 35, 55, and 75 μL. The mean relative errors decrease from 1.52% to 0.36% for ${\mathrm{SiO}}_{2}$ and from 1.47% to 0.49% for PTFE. The effective contact-line energy term, therefore, improves the average predictive accuracy over the validation set, although the improvement is not uniform at every volume. The numerical results and individual relative errors are provided in Table S1.

### 5.3. Comparison of Predicted and Experimental Droplet Profiles

**Figure 4:**
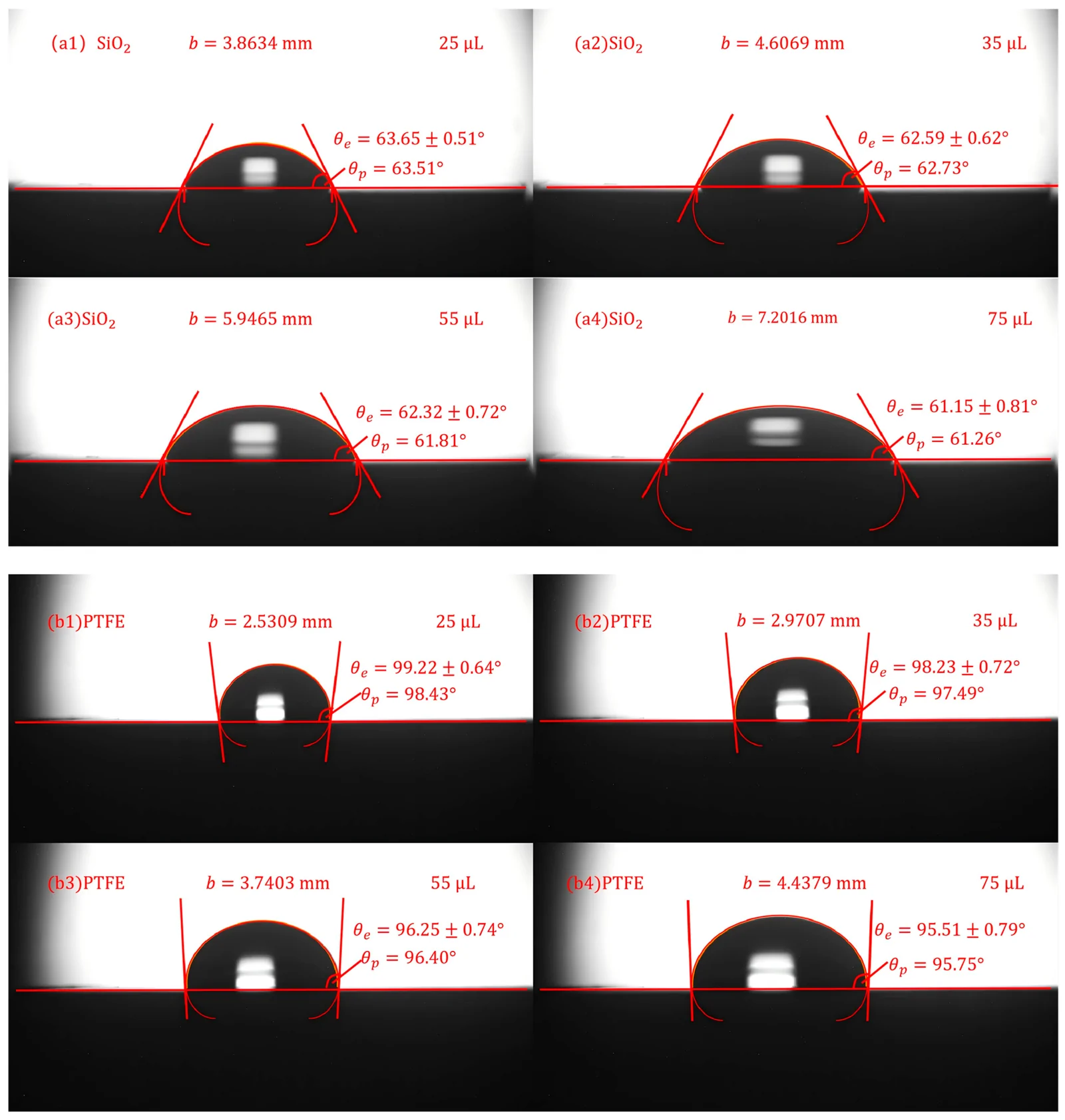
Comparisons between the predicted profiles and experimental profiles for the validation droplets: (**a1**–**a4**) droplets with volumes of 25 μL, 35 μL, 55 μL, and 75 μL on the ${\mathrm{SiO}}_{2}$ surface; (**b1**–**b4**) droplets with volumes of 25 μL, 35 μL, 55 μL, and 75 μL on the PTFE surface. The parameter b denotes the apex radius of the curvature. The symbols $\theta_{e}$ and $\theta_{p}$ denote the experimental and predicted apparent contact angles. Each panel shows a representative image selected from the five independent measurements.

To evaluate the agreement of the complete droplet shapes, the experimentally extracted profiles are compared with the corresponding theoretical predictions, as shown in Figure 4. The orange curves represent the experimentally extracted profiles, whereas the red curves represent the theoretically predicted profiles. For both surfaces and all validation volumes, the predicted and experimental profiles show close agreement over the free surface from the apex to the contact line.

### 5.4. Variation in the Apparent Contact Angle with Droplet Volume

From Equation (1), the relationship between the apparent contact angle and the contact-line radius is

$$\cos\theta_{k}=\cos\theta_{\infty }-\frac{q}{\gamma_{lv}x_{k}}$$

Differentiating this equation with respect to $x_{k}$ gives

(27) $$\frac{d\theta_{k}}{dx_{k}}=-\frac{q}{\gamma_{lv}x_{k}^{2}\sin\theta_{k}}$$

For the fitted $x_{k}>0$ and $0<\theta_{k}<\pi$, Equation (27) yields $d\theta_{k}/dx_{k}<0$. Along the calculated equilibrium branches, the contact-line radius increases with the droplet volume. The model, therefore, predicts a decrease in the apparent contact angle as the volume increases. As shown in Figure 3c,d, the experimental data exhibit the same overall trend for both the ${\mathrm{SiO}}_{2}$ and PTFE surfaces, consistent with this analysis.

## 6. Conclusions

A variational model is developed for axisymmetric sessile droplets by incorporating the surface energy, gravity, and effective contact-line excess free energy. The free surface satisfies the Bashforth–Adams equation, while the effective contact-line energy enters through the transversality condition. It, therefore, affects the equilibrium truncation point of the free surface at the substrate and results in a decrease in the apparent contact angle as the droplet volume increases. This model provides a consistent description of free-surface curvature equilibrium and effective contact-line boundary response.

The parameters q and $\theta_{\infty }$, calibrated from measurements on ${\mathrm{SiO}}_{2}$ and PTFE over 10–100 μL, are held fixed to predict droplet profiles and apparent contact angles at the excluded validation volumes of 25, 35, 55, and 75 μL. Across all validation cases, the mean relative errors in the predicted apparent contact angles are only 0.36% and 0.49% on the ${\mathrm{SiO}}_{2}$ and PTFE surfaces, respectively, with an overall mean relative error of 0.43%. The predicted profiles also show close agreement with the experimentally extracted profiles across all validation cases. These results indicate that the proposed model can reasonably predict apparent contact angles and equilibrium profiles of finite-volume droplets under different wetting conditions. The present validation was conducted within the calibration range 10–100 μL, and the extrapolation capability of the model requires further experimental assessment.

The proposed approach may be further developed into a static method for determining the effective contact-line energy parameter for a given liquid–solid system. Such a method could provide calibrated contact-line parameters for predicting droplet profiles and apparent contact angles under specified experimental conditions.

## Figures and Tables

**Table 1:** The $x_{k}$ and $\theta_{k}$ of calibration droplets on the ${\mathrm{SiO}}_{2}$ and PTFE surfaces. Values are reported as the mean ± standard deviation of five independent measurements at each volume.

Surface	Droplet Volume V/μL	Contact-Line Radius $x_{k}/\mathrm{mm}$	Apparent Contact Angle $\theta_{k}{/}^{\circ }$
${\mathrm{SiO}}_{2}$	10	1.994 ± 0.019	66.51 ± 0.52
	15	2.353 ± 0.016	64.61 ± 0.48
	20	2.604 ± 0.024	64.75 ± 0.55
	30	3.014 ± 0.032	63.28 ± 0.58
	40	3.334 ± 0.038	63.48 ± 0.57
	50	3.602 ± 0.042	62.30 ± 0.68
	70	4.240 ± 0.041	60.92 ± 0.75
	85	4.562 ± 0.049	60.80 ± 0.77
	100	4.915 ± 0.056	61.03 ± 0.84
PTFE	10	1.453 ± 0.019	102.13 ± 0.58
	15	1.709 ± 0.013	101.52 ± 0.62
	20	1.950 ± 0.019	100.03 ± 0.56
	30	2.322 ± 0.026	97.37 ± 0.69
	40	2.586 ± 0.032	97.41 ± 0.66
	50	2.863 ± 0.031	97.19 ± 0.78
	70	3.294 ± 0.042	95.96 ± 0.83
	85	3.526 ± 0.044	96.02 ± 0.88
	100	3.816 ± 0.048	94.91 ± 0.92

## Data Availability

The original contributions presented in the study are included in the article and its Supplementary Materials. Further inquiries can be directed to the corresponding author.

## Supplementary Materials

The following supporting information can be downloaded at: https://www.mdpi.com/article/10.3390/mi17101204/s1, Figure S1: Numerical procedure for predicting droplet profiles and apparent contact angles at a prescribed volume; Table S1: Experimental apparent contact angles and predictions of the calibrated model and the q=0 baseline for water droplets on ${\mathrm{SiO}}_{2}$ and PTFE surfaces at the validation volumes.
