# Supplementary material for: Sessile Droplet Profiles and Apparent Contact Angles Considering Gravity and Contact-Line Energy
Source: Micromachines (Basel). 2026 Oct 18;17(10):1204. doi: 10.3390/mi17101204 (PMC13707691; doi:10.3390/mi17101204)
Supplement: Supplementary file 1 [file micromachines-17-01204-s001.zip › micromachines-4592856-supplementary.pdf]

Sessile Droplet Profiles and Apparent Contact Angles Considering Gravity and Contact-Line Energy

Jian Dong [1,2,*], Jie Wu [1], Wenbin Fu [1] , Xuxuan Ai [1]and Chengchao Pan [3]

Manuscript micromachines-4592856

S1 Numerical formulation

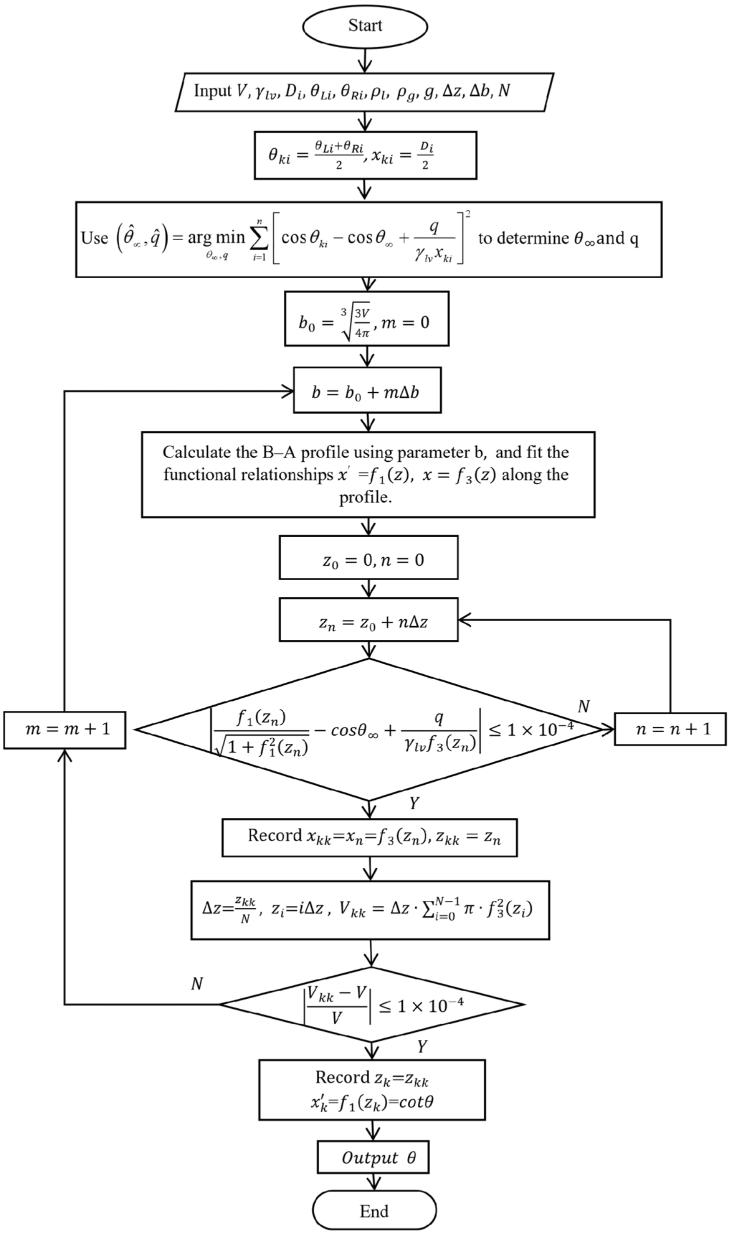

Figure S1. Numerical procedure for predicting droplet profiles and apparent contact angles at a prescribed volume.

S2. Validation results

Table S1. Experimental apparent contact angles and model predictions for water droplets on SiO_2 and PTFE surfaces at the validation volumes. Predictions include the calibrated model $(q \neq 0)$ and the $q = 0$ baseline.

Surface	Volume V(µL)	Experimental angle θ_e(°)	Model prediction $\theta_{p,q\neq0}$(°)	Baseline prediction $\theta_{p,q=0}$(°)	Relative error $q \neq 0$(%)	Relative error $q = 0$(%)
SiO_2	25	63.65±0.51	63.51	63.09	0.22	0.88
	35	62.59±0.62	62.73	63.09	0.22	0.80
	55	62.32±0.72	61.81	63.09	0.81	1.24
	75	61.15±0.81	61.26	63.09	0.18	3.17
PTFE	25	99.22±0.64	98.43	98.05	0.80	1.18
	35	98.23±0.72	97.49	98.05	0.76	0.18
	55	96.25±0.74	96.40	98.05	0.15	1.87
	75	95.51±0.79	95.75	98.05	0.25	2.66

* Experimental values are the mean ± standard deviation of five independent measurements. Relative errors are calculated from unrounded predictions as $\varepsilon_\theta = 100\left|\theta_p - \theta_e\right| / \theta_e$. Mean relative errors are the arithmetic means over the four validation volumes for each substrate. The $q = 0$ baseline angle is fitted in cosine space using the nine calibration-volume means, excluding all validation measurements. The resulting constant angles are 63.09° for SiO_2 and 98.05° for PTFE.
